# Ratio of Red Blood Cell Distribution Width to Albumin Level and Risk of Mortality

**DOI:** 10.1001/jamanetworkopen.2024.13213

**Published:** 2024-05-28

**Authors:** Meng Hao, Shuai Jiang, Jingdong Tang, Xiangnan Li, Shuming Wang, Yi Li, Jingyi Wu, Zixin Hu, Hui Zhang

**Affiliations:** 1 Department of Vascular Surgery, Shanghai Key Laboratory of Vascular Lesion Regulation and Remodeling, Shanghai Pudong Hospital, Fudan University Pudong Medical Center, Shanghai, China; 2 Greater Bay Area Institute of Precision Medicine (Guangzhou), Fudan University, Nansha District, Guangzhou, China; 3 Fudan Zhangjiang Institute, Shanghai, China; 4 Department of Macromolecular Science, State Key Laboratory of Molecular Engineering of Polymers, Fudan University, Shanghai, China; 5 Human Phenome Institute, Zhangjiang Fudan International Innovation Centre, Fudan University, Shanghai, China; 6 Artificial Intelligence Innovation and Incubation Institute, Fudan University, Shanghai, China; 7 School of Global Health, Chinese Center for Tropical Diseases Research, Shanghai Jiao Tong University School of Medicine, Shanghai, China.

## Abstract

**Importance:**

The ratio of red blood cell distribution width (RDW) to albumin concentration (RAR) has emerged as a reliable prognostic marker for mortality in patients with various diseases. However, whether RAR is associated with mortality in the general population remains unknown.

**Objectives:**

To explore whether RAR is associated with all-cause and cause-specific mortality and to elucidate their dose-response association.

**Design, Setting, and Participants:**

This population-based prospective cohort study used data from participants in the 1998-2018 US National Health and Nutrition Examination Survey (NHANES) and from the UK Biobank with baseline information provided from 2006 to 2010. Included participants had complete data on serum albumin concentration, RDW, and cause of death. The NHANES data were linked to the National Death Index records through December 31, 2019. For the UK Biobank, dates and causes of death were obtained from the National Health Service Information Centre (England and Wales) and the National Health Service Central Register Scotland (Scotland) to November 30, 2022.

**Main Outcomes and Measures:**

Potential associations between RAR and the risk of all-cause and cause-specific mortality were evaluated using Cox proportional hazards regression models. Restricted cubic spline regressions were applied to estimate possible nonlinear associations.

**Results:**

In NHANES, 50 622 participants 18 years of age or older years were included (mean [SD] age, 48.6 [18.7] years; 26 136 [51.6%] female), and their mean (SD) RAR was 3.15 (0.51). In the UK Biobank, 418 950 participants 37 years of age or older (mean [SD], 56.6 [8.1] years; 225 038 [53.7%] female) were included, and their mean RAR (SD) was 2.99 (0.31). The NHANES documented 7590 deaths over a median (IQR) follow-up of 9.4 (5.1-14.2) years, and the UK Biobank documented 36 793 deaths over a median (IQR) follow-up of 13.8 (13.0-14.5) years. According to the multivariate analysis, elevated RAR was significantly associated with greater risk of all-cause mortality (NHANES: hazard ratio [HR], 1.83 [95% CI, 1.76-1.90]; UK Biobank: HR, 2.08 [95% CI, 2.03-2.13]), as well as mortality due to malignant neoplasm (NHANES: HR, 1.89 [95% CI, 1.73-2.07]; UK Biobank: HR, 1.93 [95% CI, 1.86-2.00]), heart disease (NHANES: HR, 1.88 [95% CI, 1.74-2.03]; UK Biobank: HR, 2.42 [95% CI, 2.29-2.57]), cerebrovascular disease (NHANES: HR, 1.35 [95% CI, 1.07-1.69]; UK Biobank: HR, 2.15 [95% CI, 1.91-2.42]), respiratory disease (NHANES: HR, 1.99 [95% CI, 1.68-2.35]; UK Biobank: HR, 2.96 [95% CI, 2.78-3.15]), diabetes (NHANES: HR, 1.55 [95% CI, 1.27-1.90]; UK Biobank: HR, 2.83 [95% CI, 2.35-3.40]), and other causes of mortality (NHANES: HR, 1.97 [95% CI, 1.86-2.08]; UK Biobank: HR, 2.40 [95% CI, 2.30-2.50]) in both cohorts. Additionally, a nonlinear association was observed between RAR levels and all-cause mortality in both cohorts.

**Conclusions and Relevance:**

In this cohort study, a higher baseline RAR was associated with an increased risk of all-cause and cause-specific mortality in the general population. These findings suggest that RAR may be a simple, reliable, and inexpensive indicator for identifying individuals at high risk of mortality in clinical practice.

## Key Points

**Question:**

Is the ratio of red blood cell distribution width to albumin concentration (RAR) associated with mortality in the general population?

**Findings:**

In this cohort study involving 469 572 participants at baseline and 44 383 deaths during follow-up periods, higher RAR levels were associated with increased risks of all-cause and cause-specific mortality.

**Meaning:**

These findings suggest that the RAR, assessed via routine laboratory tests, could be a promising indicator that is simple, reliable, and inexpensive for identifying individuals at high risk of mortality in clinical practice.

## Teaser

This cohort study assesses the associations between the ratio of red blood cell distribution width to albumin concentration and all-cause and cause-specific mortality in the general population.

## Introduction

The identification of new biomarkers for the early detection of increased risk of mortality remains an unmet need. There has been significant progress in establishing the epidemiology of novel blood biomarkers for all-cause and cause-specific mortality, such as serum neurofilament light chain,^1,2^ N-terminal pro-brain natriuretic peptide,^3^ retinol,^4^ alpha tocopherol,^5^ and beta carotene.^6^ Despite this progress, simple, inexpensive, and reliable indicators are still essential for predicting mortality in clinical practice. At present, routine laboratory tests are widely used in primary care and hospitals, and further use of routine clinical biomarkers is expected. For example, several new biomarkers, such as the prognostic nutritional index, ratio of neutrophil to lymphocyte, and ratio of platelet to lymphocyte, were derived from routine laboratory tests. These simple integrated biomarkers showed significant associations with all-cause and cause-specific mortality in the general population.^7,8,9^ Therefore, there is still substantial interest in the use of routine biomarkers to identify persons who are at risk for mortality.

Red blood cell distribution width (RDW), a marker from routine laboratory tests, reflects the degree of heterogeneity in erythrocyte volume. Increased RDW, resulting from impaired erythropoiesis and abnormal red blood cell survival, are associated with a variety of disorders and mortality.^10^ Serum albumin, the most abundant circulating protein in the blood, is an important marker of nutritional status and the inflammatory response.^11,12^ The physiological properties of albumin include anti-inflammatory, antioxidant, anticoagulant, and antiplatelet aggregation activities as well as colloid osmotic effects.^13^ Several studies have reported that the serum albumin concentration is inversely associated with the incidence of dysfunction, disease, and mortality.^14,15,16^ Additionally, RDW is positively correlated with chronological age, while serum albumin is negatively correlated with chronological age,^17^ and RDW and albumin are associated with phenotypic and biological age and contribute greatly to individual variances in aging pace.^18,19,20^

Both RDW and the albumin concentration have been suggested as integrative biomarkers for a multidimensional dysfunctional physiological status that relates to inflammation, oxidative stress, and nutrition,^10,11,12^ but they represent these pathological aspects from different perspectives. Therefore, considering their essential roles in physiological function, disease, and assessment of aging, the integration of these 2 markers may be valuable for predicting mortality. The ratio of RDW to albumin concentration (RAR), a marker derived from routine laboratory tests, may convey large amounts of information beyond either value alone. Most recently, RAR has emerged as a potential risk biomarker for adverse outcomes in various diseases, including acute myocardial infarction,^21^ atrial fibrillation,^22^ diabetes,^23^ heart failure,^24^ chronic kidney disease,^25^ and stroke.^26^ However, whether RAR is associated with mortality in the general population remains unknown.

To address this knowledge gap, we first hypothesized that elevated RAR is associated with increased risk of mortality in the general population and then examined the potential association bnonetween RAR levels and the risk of all-cause and cause-specific mortality using data from the US National Health and Nutrition Examination Survey (NHANES) and the UK Biobank.

## Methods

### Study Population

The US NHANES is a nationally representative cross-sectional survey of civilian, noninstitutionalized persons living in the US. The present cohort study used data from NHANES 1998 through 2018. The data included demographic information, physical examination results, questionnaire items, and mortality status linked to the National Death Index. In this cohort, we also used data from a publicly available data dictionary,^27^ and participants aged 18 years or older with complete data on serum albumin concentration, RDW, and death were included and analyzed. The final sample from NHANES included 50 622 (aged 19-85 years) individuals (eFigure 1 in Supplement 1). Institutional review board approval was waived for this analysis because of the publicly available and deidentified data. The UK Biobank is a large-scale, population-based, prospective cohort study consisting of more than 500 000 adults (aged 37-73 years). During the baseline period (2006 to 2010), information about participants was collected through questionnaires, physical measurements, and biological samples. All participants provided electronic informed consent. The UK Biobank study received approval from the National Information Governance Board for Health and Social Care and the National Health Service North West Multi-Centre Research Ethics Committee. In this cohort, a total of 418 950 participants with complete data on serum albumin concentration, RDW, and mortality, including cause of death, were analyzed (eFigure 1 in Supplement 1). Additionally, we added information on the excluded population and tested the differences between the included and excluded populations (eTable 1 and eTable 2 in Supplement 1). This study followed the Strengthening the Reporting of Observational Studies in Epidemiology (STROBE) reporting guideline for cohort studies.

### Measurement of RAR

In NHANES, the serum albumin concentration was determined using the bromocresol purple method. The RDW (percentage) was measured by a Coulter analyzer in the mobile examination centers using peripheral blood. In the UK Biobank, the serum albumin concentration was measured using a colorimetric approach and a Beckman Coulter AU5800 assay (Beckman Coulter). The RDW (percentage) was measured using 4 Beckman Coulter LH750 instruments within 24 hours of blood collection, with extensive quality control performed by the UK Biobank. In our study, the independent variable was RAR.

### Outcomes

In this study, the primary outcome was all-cause mortality. The secondary outcome was cause-specific mortality. In the US NHANES, the dates and causes of death were linked to the National Death Index records through December 31, 2019. In the UK Biobank, dates and causes of death were obtained from the National Health Service Information Centre (England and Wales) and the National Health Service Central Register Scotland (Scotland) to November 30, 2022. We used the *International Statistical Classification of Diseases, Tenth Revision*, to identify causes of death. The mortality outcomes in this study included the following underlying causes of death: malignant neoplasm, heart disease, cerebrovascular disease, respiratory disease, Alzheimer disease, and others.

### Covariates

In this study, covariates included demographic characteristics, lifestyle factors, and clinical information. The demographic characteristics included age at baseline, sex, race and ethnicity, and education. Race and ethnicity were assessed because they may play important roles in the association between RAR and mortality. Lifestyle factors included body mass index (BMI; calculated as weight in kilograms divided by height in meters squared), smoking status (ever or never), and alcohol consumption (yes or no). The BMI was used to divide participants into underweight (BMI, <18.5), normal weight (BMI, 18.5-24.9), overweight (BMI, 25.0-29.9), and obese (BMI, ≥30.0) groups. Information on demographic characteristics and lifestyle factors was collected through a self-report questionnaire. In the UK Biobank, race and ethnicity status was categorized as White and other race and ethnicity. The White group included British, Irish, and any other White background; other race and ethnicity included Asian or Asian British (including Bangladeshi, Indian, Pakistani, and any other Asian background), Black or Black British (including African, Caribbean, and any other Black background), Chinese, mixed race (including Asian and White, Black and White African, Black and White Caribbean, and any other mixed background), or other ethnic group. In NHANES, race and ethnicity status was categorized into Black (non-Hispanic Black), Hispanic (Mexican American and other Hispanic), White (non-Hispanic White), and other (Asian, multiple races). Educational levels were divided into college or above, high school or equivalent, and less than high school. In the UK Biobank, participants who reported their education qualifications as college or university degree were categorized into the group with college or above. Participants who reported advanced level, advanced subsidiary level, or equivalent; ordinary level, general certificate of secondary education or equivalent; certificate of secondary education or equivalent; national vocational qualification, higher national diploma, higher national certificate, or equivalent; or other professional qualifications were categorized into the high school or equivalent group. Participants who reported none of the above were categorized into the less than high school group.^28^ In NHANES, participants reported their education qualifications as college graduate or above, some college or associate of arts degree, high school graduate or General Educational Development or equivalent, 9th through 11th grade (including 12th grade with no diploma), less than 9th grade, and unknown or missing (excluded from our analyses). Clinical information, including the presence of hypertension, diabetes, heart disease, stroke, and cancer, was obtained from self-reports by participants or proxies.

### Statistical Analysis

First, continuous and categorical data are presented as means and SDs or frequencies (percentages). Second, we used Cox proportional hazards regression models to estimate the hazard ratios (HRs) and 95% CIs of continuous RAR levels associated with all-cause and cause-specific mortality using 2 models: model 1 was the crude model, and model 2 was adjusted for age (continuous), sex, education, race and ethnicity, smoking status, alcohol consumption status, BMI (continuous), hypertension, diabetes, heart disease, stroke, and cancer. Third, subgroups were created according to age group (<45, 45-64, and ≥65 years), sex, BMI category, race and ethnicity, educational level, smoking status, and alcohol consumption status. A stratified analysis was conducted to examine the association between RAR levels and all-cause mortality in model 2. The *P* values for interactions were evaluated through likelihood ratio tests by comparing Cox proportional hazards regression models with or without the cross-product terms for each assessed factor. Fourth, we conducted restricted cubic spline regressions to estimate possible nonlinearity between RAR and the risk of all-cause and cause-specific mortality after adjusting for confounding factors (model 2). Finally, we categorized the RAR levels into 4 quartiles (Qs; for NHANES: Q1, 2.02-2.82; Q2, >2.82-3.05; Q3, >3.05-3.35; and Q4, >3.35-12.10; for the UK Biobank: Q1, 0.46-2.80; Q2, >2.80-2.96; Q3, >2.96-3.14; and Q4, >3.14-10.10) and then examined the associations between the RAR groups and the risk of mortality using model 2. Additionally, tests for trends across the 4 groups were conducted. We also conducted a Kaplan-Meier survival analysis to plot the associations of different RAR groups with all-cause and cause-specific mortality. All results were considered significant at *P* < .05 (2-tailed). All analyses were conducted using R statistical software, version 4.2.1 (R Project for Statistical Computing).

## Results

### Characteristics of the Study Population

Table 1 shows the characteristics of the study population in NHANES and the UK Biobank. The baseline demographic characteristics, lifestyle factors, and chronic diseases of the study population are presented in detail. At baseline, 50 622 participants (44.9% <45 years; 51.6% female and 48.4% male; 20.5% Black, 26.4% Hispanic, 44.0% White, and 9.1% other ethnicity) in NHANES were analyzed, and 418 950 participants (10.2% <45 years; 53.7% female and 46.3% male; 94.3% White ethnicity and 5.7% other race and ethnicity) in the UK Biobank were analyzed. The mean (SD) ages of the participants were 48.6 (18.7) years in NHANES and 56.6 (8.1) years in the UK Biobank. The mean (SD) RAR levels were 3.15 (0.51) in NHANES and 2.99 (0.31) in the UK Biobank. In addition, NHANES documented 7590 deaths over a median (IQR) follow-up of 9.4 (5.1-14.2) years, and the UK Biobank documented 36 793 deaths over a median (IQR) follow-up of 13.8 (13.0-14.5) years.

**Table 1.** Baseline Characteristics of the Study Population

Characteristic	Participants, No. (%)	
	NHANES (N = 50 622)	UK Biobank (N = 418 950)
Age, mean (SD), y	48.6 (18.7)	56.6 (8.1)
Age category		
<45	22 716 (44.9)	42 897 (10.2)
45-64	15 878 (31.4)	295 704 (70.6)
≥65	12 028 (23.8)	80 349 (19.2)
Sex		
Female	26136 (51.6)	225 038 (53.7)
Male	24 486 (48.4)	193 912 (46.3)
Race and ethnicity^a^		
Black	10 366 (20.5)	NA
Hispanic	13 383 (26.4)	NA
White	22 277 (44.0)	395 095 (94.3)
Other	4596 (9.1)	23 855 (5.7)
Educational status		
≥College or above	24 331 (48.0)	135 232 (32.3)
High school or equivalent	11 270 (22.2)	207 086 (49.5)
<High school	13 181 (26.0)	71 628 (17.1)
Alcohol consumption (yes)	32 792 (66.6)	384 483 (91.9)
Smoking status (ever)	22 369 (45.4)	189 097 (45.2)
BMI, mean (SD)	28.86 (6.8)	27.43 (4.8)
BMI category		
Underweight (<18.5)	847 (1.7)	2141 (0.5)
Normal (18.5-24.9)	14 311 (28.8)	135 349 (32.4)
Overweight (25.0-29.9)	16 701 (33.6)	177 934 (42.6)
Obese (≥30.0)	17 888 (36.0)	101 857 (24.4)
RDW, mean (SD), %	13.17 (1.3)	13.49 (1.0)
Albumin, mean (SD), g/dL	4.23 (0.37)	4.52 (0.26)
RAR, mean (SD)	3.15 (0.51)	2.99 (0.31)
Chronic disease		
Hypertension	16 969 (33.5)	100 408 (24.0)
Diabetes	5896 (11.7)	21 821 (5.2)
Heart disease	3808 (7.5)	19 061 (4.6)
Stroke	1835 (3.6)	5114 (1.2)
Cancer	4423 (8.7)	31 583 (7.6)

Abbreviations: BMI, body mass index (calculated as weight in kilograms divided by height in meters squared); NA, not applicable; NHANES, National Health and Nutrition Examination Survey; RAR, ratio of red blood cell distribution width to albumin concentration; RDW, red blood cell distribution width.

SI conversion factor: To convert albumin to grams per liter, multiply by 10.

^a^ In NHANES, other included Asian and multiple races. In the UK Biobank, other included Asian or Asian British (including Bangladeshi, Indian, Pakistani, and any other Asian background), Black or Black British (including African, Caribbean, and any other Black background), Chinese, mixed race (including Black and White African, Black and White Caribbean, Asian and White, any other mixed background), or other ethnic group.

### Association Between RAR and Mortality

The results of Cox proportional hazards regression analyses regarding the association of RAR with the risk of all-cause and cause-specific mortality are presented in Table 2. In NHANES, after adjusting for covariates, elevated RAR levels were associated with increased risk of all-cause mortality (HR, 1.83 [95% CI, 1.76-1.90]) and disease-specific causes of mortality: malignant neoplasm (HR, 1.89 [95% CI, 1.73-2.07]), heart disease (HR, 1.88 [95% CI, 1.74-2.03]), cerebrovascular disease (HR, 1.35 [95% CI, 1.07-1.69]), respiratory disease (HR, 1.99 [95% CI, 1.68-2.35]), Alzheimer disease (HR, 1.33 [0.98-1.81]), diabetes (HR, 1.55 [95% CI, 1.27-1.90]), and other causes of mortality (HR, 1.97 [95% CI, 1.86-2.08]). In the UK Biobank, similar positive associations were also found between RAR levels and all-cause mortality (HR, 2.08 [95% CI, 2.03-2.13]) and disease-specific causes of mortality: malignant neoplasm (HR, 1.93 [95% CI, 1.86-2.00]), heart disease (HR, 2.42 [95% CI, 2.29-2.57]), cerebrovascular disease (HR, 2.15 [95% CI, 1.91-2.42]), respiratory disease (HR, 2.96 [95% CI, 2.78-3.15]), diabetes (HR, 2.83 [95% CI, 2.35-3.40]), and other causes of mortality (HR, 2.40 [95% CI, 2.30-2.50]).

**Table 2.** Hazard Ratios of Mortality According to Continuous Levels of Ratio of Red Blood Distribution Width to Albumin

Population	No. of events	Model 1^a^		Model 2^b^	
		HR (95% CI)	*P* value	HR (95% CI)	*P* value
**NHANES**					
All-cause mortality	7590	2.01 (1.96-2.07)	<.001	1.83 (1.76-1.90)	<.001
Cause-specific mortality					
Malignant neoplasm	1638	1.97 (1.84-2.10)	<.001	1.89 (1.73-2.07)	<.001
Heart disease	1961	2.17 (2.05-2.29)	<.001	1.88 (1.74-2.03)	<.001
Cerebrovascular disease	433	1.88 (1.64-2.15)	<.001	1.35 (1.07-1.69)	.01
Respiratory disease	412	2.07 (1.82-2.35)	<.001	1.99 (1.68-2.35)	<.001
Alzheimer disease	283	1.70 (1.41-2.06)	<.001	1.33 (0.98-1.81)	.07
Diabetes	277	2.21 (1.91-2.55)	<.001	1.55 (1.27-1.90)	<.001
Other cause	2586	2.13 (2.05-2.23)	<.001	1.97 (1.86-2.08)	<.001
**UK Biobank**					
All-cause mortality	36 793	2.32 (2.28-2.36)	<.001	2.08 (2.03-2.13)	<.001
Cause-specific mortality					
Malignant neoplasm	17 657	2.14 (2.07-2.20)	<.001	1.93 (1.86-2.00)	<.001
Heart disease	5358	2.57 (2.46-2.68)	<.001	2.42 (2.29-2.57)	<.001
Cerebrovascular disease	1623	2.36 (2.16-2.57)	<.001	2.15 (1.91-2.42)	<.001
Respiratory disease	2636	2.84 (2.71-2.98)	<.001	2.96 (2.78-3.15)	<.001
Alzheimer disease	687	1.32 (1.05-1.66)	.02	0.85 (0.64-1.14)	.28
Diabetes	253	3.18 (2.82-3.59)	<.001	2.83 (2.35-3.40)	<.001
Other cause	8579	2.51 (2.43-2.59)	<.001	2.40 (2.30-2.50)	<.001

Abbreviations: HR, hazard ratio; NHANES, National Health and Nutrition Examination Survey.

^a^ Model 1 is the crude model.

^b^ Model 2 is adjusted for age, sex, body mass index, race, educational level, smoking status, alcohol consumption, hypertension, diabetes, heart disease, stroke, and cancer.

**Figure 1.**
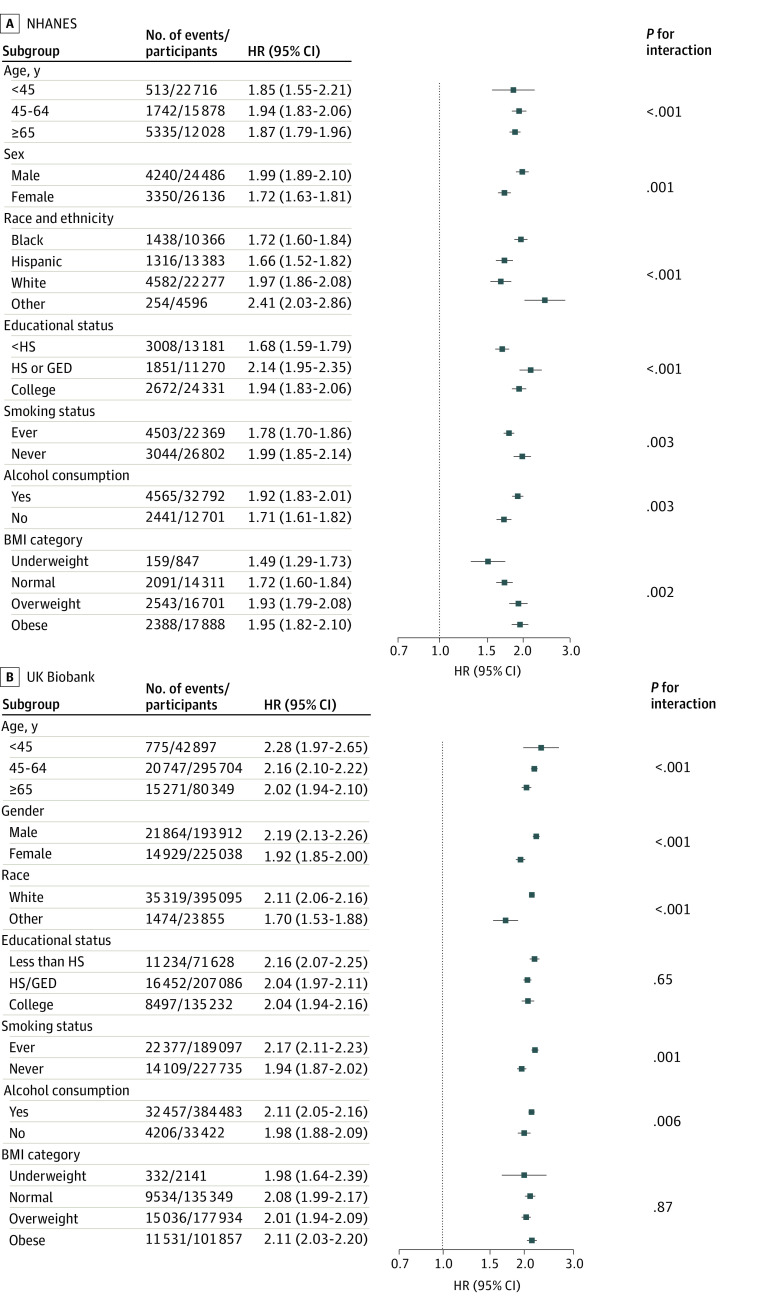
Associations Between Ratio of Red Blood Distribution Width to Albumin and All-Cause Mortality Among Subgroups All models were adjusted for age, sex, body mass index (BMI), race, educational level, smoking status, alcohol consumption, hypertension, diabetes, heart disease, stroke, and cancer. In NHANES, other race and ethnicity included Asian and multiple races. In the UK Biobank, other included Asian or Asian British (including Bangladeshi, Indian, Pakistani, and any other Asian background), Black or Black British (including African, Caribbean, and any other Black background), Chinese, mixed race (including Black and White African, Black and White Caribbean, Asian and White, any other mixed background), or other ethnic group. The *P* value for the interaction was calculated using the likelihood ratio test. GED indicates General Educational Development; HR, hazard ratio; HS, high school; and NHANES, National Health and Nutrition Examination Survey.

**Figure 2.**
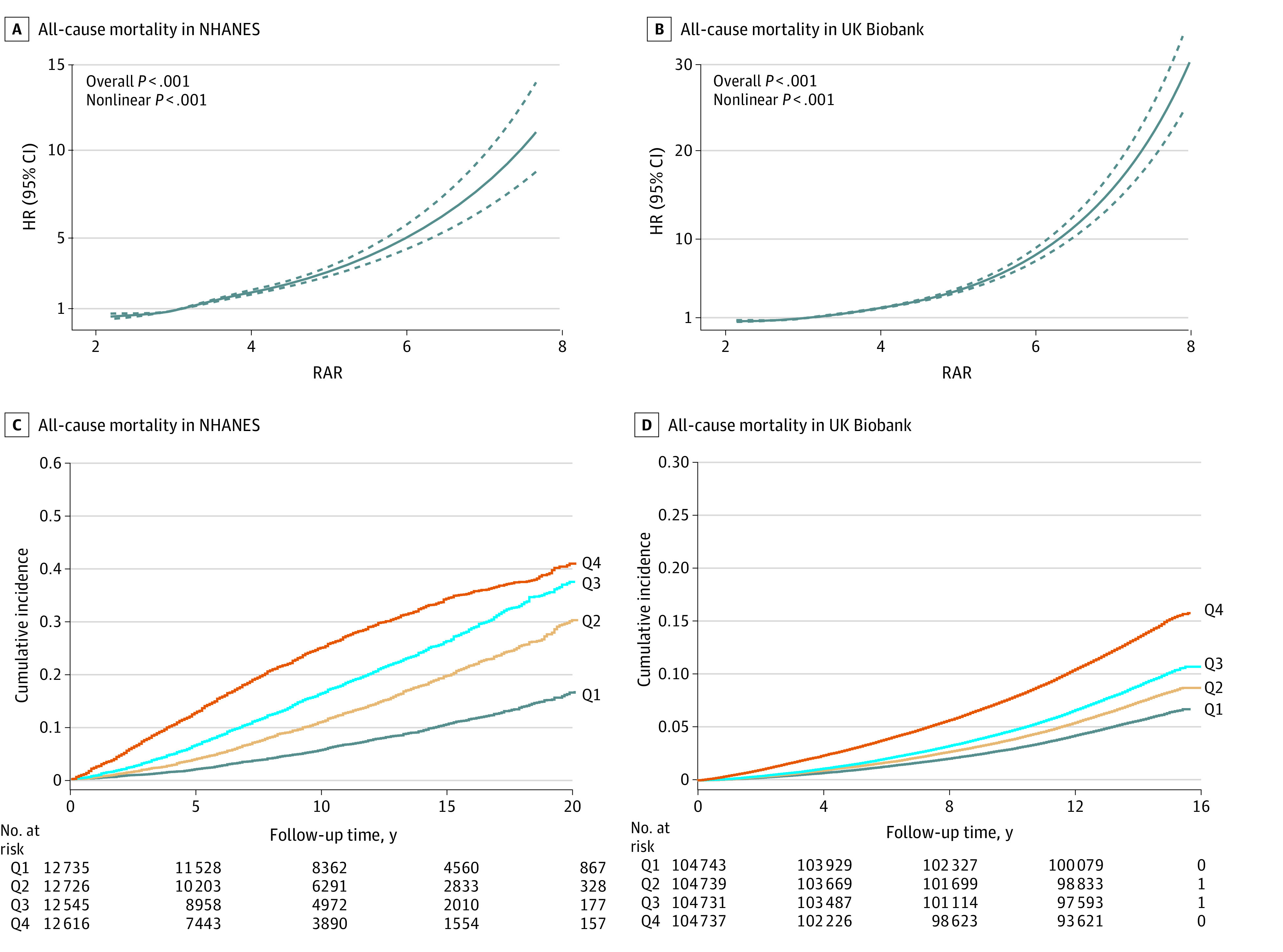
Association Between Baseline RAR and Future Risk of Mortality Cubic spline regression for estimated hazard ratios of all-cause mortality according to continuous levels of the ratio of red blood cell distribution width to albumin concentration (RAR) in the National Health and Nutrition Examination Survey (NHANES) (A) and the UK Biobank (B). All models were adjusted for age, sex, body mass index, race, educational level, smoking status, alcohol consumption, hypertension, diabetes, heart disease, stroke, and cancer. Cumulative incidence of all-cause mortality by RAR quartile in NHANES (C) and the UK Biobank (D).Dotted lines in A represent the 95% CIs. HR indicates hazard ratio; Q, RAR quartile.

In addition, we conducted stratified analyses of the 2 cohorts by age group, sex, BMI category, race and ethnicity, and smoking and alcohol consumption status (Figure 1). We found significant associations between elevated RAR levels and increased risk of all-cause mortality in each stratum (eg, in the UK Biobank for age group <45 years, HR, 2.28 [95% CI, 1.97-2.65]; 45-64 years, 2.16 [95% CI, 2.10-2.22]; and ≥65 years of age: 2.02 [95% CI, 1.94-2.10]; *P* < .001 for interaction). These findings suggest that elevated RAR levels are associated with an increased risk of all-cause mortality, regardless of socioeconomic status, lifestyle factors, chronic diseases, and mental health. We also used cubic spline regression to evaluate the RAR-mortality dose-response association, modeling RAR as a continuous variable. We found a nonlinear, positive association between RAR and all-cause mortality (Figure 2A and B) in both cohorts (*P* < .001 for nonlinearity). Cubic spline regression was used to evaluate the dose-response association between RAR and cause-specific mortality (eFigure 2 in Supplement 1).

In further analyses, we categorized the RAR levels into 4 groups based on quartiles in NHANES and the UK Biobank and then examined the associations between the RAR groups and the risk of mortality (Table 3). Compared with participants in the lowest quartile of RAR, those in higher quartiles experienced significantly greater mortality from all causes (eg, quartile 4 NHANES: HR, 2.20 [95% CI, 2.02-2.40]; *P* < .001 and quartile 4 UK Biobank: HR, 1.82 [95% CI, 1.76-1.88]; *P* < .001), malignant neoplasm, heart disease, cerebrovascular disease, respiratory disease, diabetes, and other causes, representing a 10% to 369% increase in risk (all *P* < .001 for trend). Kaplan-Meier survival plots indicated that participants in the higher RAR quartiles had significantly greater cumulative all-cause mortality than those in the lowest quartiles (Figure 2C and D). Similar patterns were observed for cause-specific mortality (eFigure 3 in Supplement 1).

**Table 3.** Hazard Ratios for Mortality, by Ratio of Red Blood Distribution Width to Albumin Quartile^a^

Mortality cause	NHANES			UK Biobank		
	No. of events	HR (95% CI)	*P* value	No. of events	HR (95% CI)	*P* value
**All causes**						
Q1	1146	1 [Reference]	NA	5819	1 [Reference]	NA
Q2	1732	1.16 (1.07-1.26)	<.001	7622	1.13 (1.09-1.17)	<.001
Q3	2072	1.42 (1.30-1.54)	<.001	9276	1.26 (1.22-1.30)	<.001
Q4	2640	2.20 (2.02-2.40)	<.001	14 076	1.82 (1.76-1.88)	<.001
*P* value for trend	NA	NA	<.001	NA	NA	<.001
**Malignant neoplasm**						
Q1	280	1 [Reference]	NA	3055	1 [Reference]	NA
Q2	389	1.06 (0.90-1.25)	.50	3857	1.10 (1.05-1.16)	<.001
Q3	465	1.31 (1.10-1.55)	.01	4529	1.20 (1.15-1.26)	<.001
Q4	504	1.85 (1.54-2.21)	<.001	6216	1.63 (1.56-1.71)	<.001
*P* value for trend	NA	NA	<.001	NA	NA	<.001
**Heart disease**						
Q1	263	1 [Reference]	NA	735	1 [Reference]	NA
Q2	427	1.13 (0.96-1.33)	.14	1027	1.23 (1.11-1.35)	<.001
Q3	540	1.38 (1.17-1.63)	<.001	1352	1.48 (1.35-1.62)	<.001
Q4	731	2.22 (1.87-2.63)	<.001	2244	2.31 (2.12-2.53)	<.001
*P* value for trend	NA	NA	<.001	NA	NA	<.001
**Cerebrovascular disease**						
Q1	70	1 [Reference]	NA	251	1 [Reference]	NA
Q2	122	1.09 (0.79-1.50)	.60	345	1.19 (1.01-1.41)	.04
Q3	113	1.05 (0.74-1.48)	.79	420	1.31 (1.11-1.54)	<.001
Q4	128	1.31 (0.89-1.92)	.17	607	1.80 (1.54-2.11)	<.001
*P* value for trend	NA	NA	.28	NA	NA	<.001
**Respiratory disease**						
Q1	54	1 [Reference]	NA	272	1 [Reference]	NA
Q2	95	1.43 (1.00-2.04)	.02	428	1.37 (1.17-1.60)	<.001
Q3	126	2.03 (1.42-2.90)	<.001	659	1.87 (1.62-2.16)	<.001
Q4	137	3.20 (2.18-4.69)	<.001	1277	3.50 (3.06-4.02)	<.001
*P* value for trend	NA	NA	<.001	NA	NA	<.001
**Alzheimer disease**						
Q1	52	1 [Reference]	NA	150	1 [Reference]	NA
Q2	73	0.81 (0.55-1.19)	.29	162	0.83 (0.66-1.04)	.11
Q3	91	1.18 (0.80-1.74)	.40	181	0.83 (0.66-1.03)	.10
Q4	67	1.17 (0.74-1.84)	.50	194	0.87 (0.69-1.08)	.20
*P* value for trend	NA	NA	.27	NA	NA	.25
**Diabetes**						
Q1	43	1 [Reference]	NA	22	1 [Reference]	NA
Q2	40	0.73 (0.46-1.16)	.18	39	1.71 (0.99-2.94)	.06
Q3	82	1.35 (0.88-2.07)	.17	55	2.26 (1.33-3.83)	.003
Q4	112	2.25 (1.46-3.48)	<.001	137	4.69 (2.88-7.64)	<.001
*P* value for trend	NA	NA	<.001	NA	NA	<.001
**Other**						
Q1	384	1 [Reference]	NA	1334	1 [Reference]	NA
Q2	586	1.27 (1.11-1.47)	<.001	1764	1.15 (1.07-1.23)	<.001
Q3	655	1.48 (1.31-1.76)	<.001	2080	1.25 (1.16-1.34)	<.001
Q4	961	2.66 (2.29-3.08)	<.001	3401	2.01 (1.88-2.15)	<.001
*P* value for trend	NA	NA	<.001	NA	NA	<.001

Abbreviations: HR, hazard ratio; NA, not applicable; NHANES, National Health and Nutrition Examination; Q1 through Q4, quartile 1 through 4.

^a^ All models are adjusted for age, sex, body mass index, race, educational levels, smoking status, alcohol consumption, hypertension, diabetes, heart disease, stroke, and cancer.

## Discussion

To our knowledge, this cohort study is the first and largest study in which the association of RAR with all-cause and cause-specific mortality was examined. Using data from NHANES and the UK Biobank, involving approximately 470 000 participants at baseline and 44 383 deaths during follow-up periods, we found that higher RAR levels were independently associated with increased risks of all-cause and cause-specific mortality in the general population. The restricted cubic spline analysis also indicated increasing all-cause and cause-specific mortality risks with increasing RAR levels. Hence, RAR may be a novel and promising biomarker for identifying individuals at high risk of mortality in clinical practice.

Previous studies have examined the association of RAR with mortality in various disease-specific populations, such as patients with acute myocardial infarction,^21^ atrial fibrillation,^22^ diabetes,^23^ heart failure,^24^ stroke,^26^ burn surgery,^29^ and cervical cancer.^30^ For example, Hong et al^23^ included 860 patients with diabetic foot ulcers in China and found a positive association between a high RAR and all-cause mortality (HR, 2.36 [95% CI, 1.41-3.94) after adjusting for confounding factors. Zhao et al^26^ included 1480 patients with stroke from the Medical Information Mart for Intensive Care III database and reported that patients in the highest quartile of RAR had the highest 30-day (HR, 1.88 [95% CI, 1.36-2.58]), 90-day (HR, 2.12 [95% CI, 1.59-2.82]), and 1-year (HR, 2.15 [95% CI, 1.65-2.80]) all-cause mortality compared with patients in the first quartile. These studies focused on various disease-specific populations and indicated significant associations of RAR with adverse outcomes. Compared with the results of previous studies, our study is the first to investigate RAR and mortality in the general population.

In addition, RAR has also emerged as a reliable prognostic marker for other adverse outcomes in various diseases. Kimura et al^25^ conducted a retrospective cohort study of 997 patients with chronic kidney disease who were enrolled in the Fukushima Cohort Study. They found that patients in the highest tertile of RAR had a significantly greater risk of end-stage kidney disease (HR, 2.92 [95% CI, 1.44-5.94]), all-cause mortality (HR, 3.38 [95% CI, 1.81-6.30]), and cardiovascular events (HR, 2.27 [95% CI, 1.36-3.78]) than those in the lowest tertile of RAR. Huang et al^31^ included and analyzed 10 267 patients with congenital heart disease in China, and found that an elevated RAR was significantly associated with an increased risk of carotid plaque formation (odds ratio, 1.23 [95% CI, 1.08-1.39]).^31^ In summary, previous studies have indicated that RAR is relevant to the development of cardiovascular disease, kidney disease, and other chronic diseases. Therefore, we examined the association of RAR levels with cause-specific mortality in the NHANES and UK Biobank cohorts. Our findings provide strong epidemiologic evidence that elevated RAR levels are associated with an increased risk of cause-specific mortality in the general population.

The biological mechanisms underlying the association between higher RAR and mortality risk are unclear. These findings are likely due to chronic inflammation and nutritional status. Chronic inflammation and nutritional status play important roles in the development of mortality^7,32^ and various chronic diseases, including cardiovascular disease, cancer, diabetes, chronic kidney disease, and neurodegenerative disorders.^33^ The RDW reflects the degree of heterogeneity of erythrocyte volume. Increased RDW values can mirror impaired erythropoiesis and abnormal red blood cell survival through a variety of underlying metabolic abnormalities, such as inflammation and poor nutritional status.^10^ Serum albumin concentration is an important marker of nutritional status and the inflammatory response.^11,12^ Previous studies have reported that greater RDW^34,35,36,37^ and lower serum albumin concentration^14,15,16^ are both associated with an increased risk of incident chronic disease and mortality in the general population. Therefore, we hypothesized that the integration of these 2 markers may reflect inflammation, malnutrition, and other abnormalities throughout the lifespan and could be associated with all-cause and cause-specific mortality. Our study findings support the use of RAR as a derived biomarker in routine laboratory tests in practice and provided insights into the integration of common routine biomarkers.

### Strengths and Limitations

Our study has several strengths. We conducted this study in 2 large, prospective, population-based cohorts with long follow-up periods for cause-specific mortality. We were able to obtain relatively robust results with a large population, and the findings of our study may be relatively generalizable given that we assessed general populations.

Several limitations of the study should be noted. First, this was an observational study, and the associations of RAR with all-cause and cause-specific mortality show associations rather than causality. Second, the levels of RAR were assessed only at 1 time point, and their potential trajectories were not accounted for in this study. A single measurement of RAR may lead to some misclassification in these 2 populations. Further studies with repeated measurements of RAR should be conducted to determine its trajectory, and our findings should be validated in the future. Finally, although we controlled for many potential confounders, including demographic factors, chronic diseases, and lifestyles, we cannot rule out all potential residual confounders, especially unmeasured variables.

## Conclusions

This cohort study found that the RAR, which is derived from blood RDW and serum albumin concentrations, was strongly and independently associated with an increased risk of all-cause mortality, as well as mortality due to malignant neoplasm, heart disease, cerebrovascular disease, respiratory disease, diabetes, and others in the general population. Additionally, since RAR is assessed via routine laboratory tests, it could be recognized as a promising indicator that is simple, reliable, and inexpensively accessible for identifying individuals in clinical practice at high risk of mortality. Future studies are needed to validate these findings and investigate the potential relationship between RAR and all-cause mortality risk.

## Notes

Supplement 1. **eFigure 1.** Flowchart of study population in NHANES and the UK Biobank **eFigure 2.** Cubic spline regression for estimated hazard ratios of cause-specific mortality according to continuous levels of RAR in NHANES and the UK Biobank **eFigure 3.** Cumulative incidence of all-cause mortality according to quartiles of RAR in NHANES and the UK Biobank **eTable 1.** Baseline characteristics of included and excluded population in NHANES **eTable 2.** Baseline characteristics of included and excluded population in UK Biobank

Supplement 2.
Data Sharing Statement
