# Supplementary material for: Ratio of Red Blood Cell Distribution Width to Albumin Level and Risk of Mortality
Source: JAMA Netw Open. 2024 May 28;7(5):e2413213. doi: 10.1001/jamanetworkopen.2024.13213 (PMC11134218; doi:10.1001/jamanetworkopen.2024.13213)
Supplement: Supplement 1. — eFigure 1. Flowchart of study population in NHANES and the UK Biobank eFigure 2. Cubic spline regression for estimated hazard ratios of cause-specific mortality according to continuous levels of RAR in NHANES and the UK Biobank eFigure 3. Cumulative incidence of all-cause mortality according to quartiles of RAR in NHANES and the UK Biobank eTable 1. Baseline characteristics of included and excluded population in NHANES eTable 2. Baseline characteristics of included and excluded population in UK Biobank [file jamanetwopen-e2413213-s001.pdf]

Supplementary Online Content

Hao M, Jiang S, Tang J, et al. Ratio of red blood cell distribution width to albumin level and risk of mortality. *JAMA Netw Open*. 2024;7(5):e2413213. doi:10.1001/jamanetworkopen.2024.13213

eFigure 1. Flowchart of study population in NHANES and the UK Biobank

eFigure 2. Cubic spline regression for estimated hazard ratios of cause-specific mortality according to continuous levels of RAR in NHANES and the UK Biobank

eFigure 3. Cumulative incidence of all-cause mortality according to quartiles of RAR in NHANES and the UK Biobank

eTable 1. Baseline characteristics of included and excluded population in NHANES

eTable 2. Baseline characteristics of included and excluded population in UK Biobank

eTable 3. Statistics of associations between RAR (continuous) and mortality

eTable 4. Statistics of associations between RAR (quartiles) and mortality in NHANES

eTable 5. Statistics of associations between RAR (quartiles) and mortality in UK Biobank

This supplementary material has been provided by the authors to give readers additional information about their work.

© 2024 Hao M et al. *JAMA Network Open*.

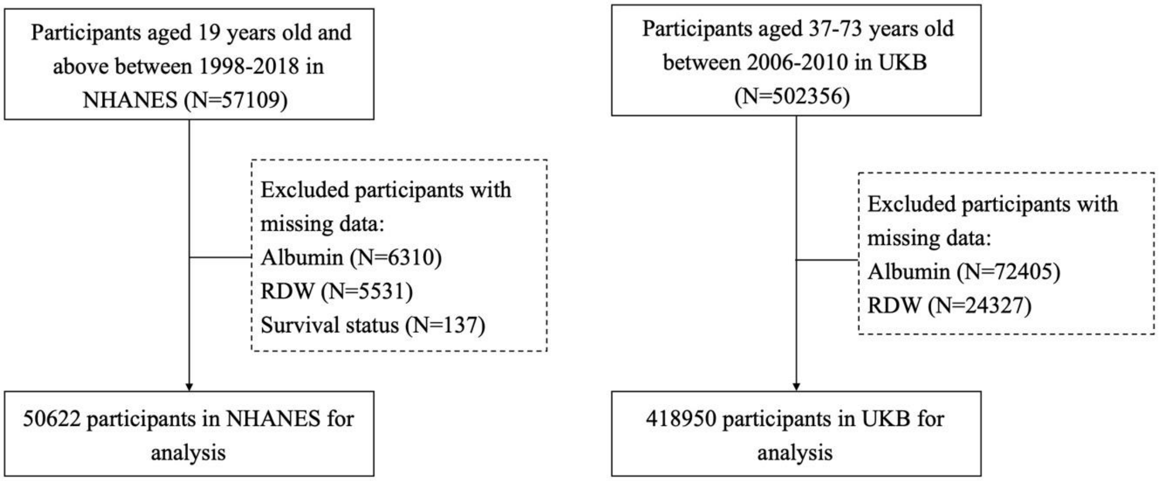

eFigure 1. Flowchart of study population in NHANES and the UK Biobank

© 2024 Hao M et al. *JAMA Network Open*.

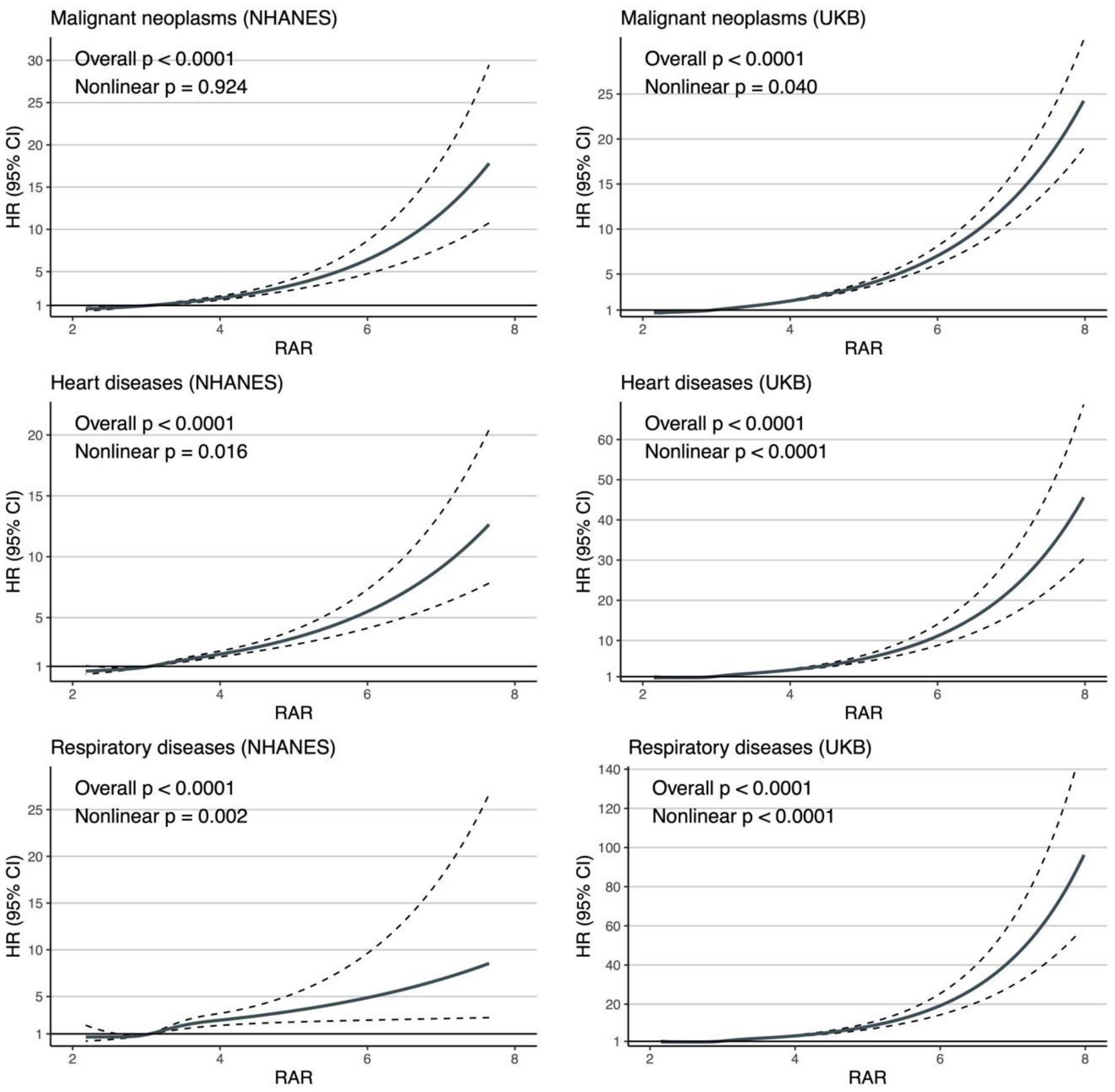

eFigure 2. Cubic spline regression for estimated hazard ratios of cause-specific mortality according to continuous levels of RAR in NHANES and the UK Biobank. All models were adjusted for age, gender, body mass index, race, educational level, smoking status, alcohol consumption, hypertension, diabetes, heart disease, stroke, and cancer. RAR: ratio of red cell distribution width to albumin; HR: hazard ratio; CI: confidence interval.

© 2024 Hao M et al. *JAMA Network Open*.

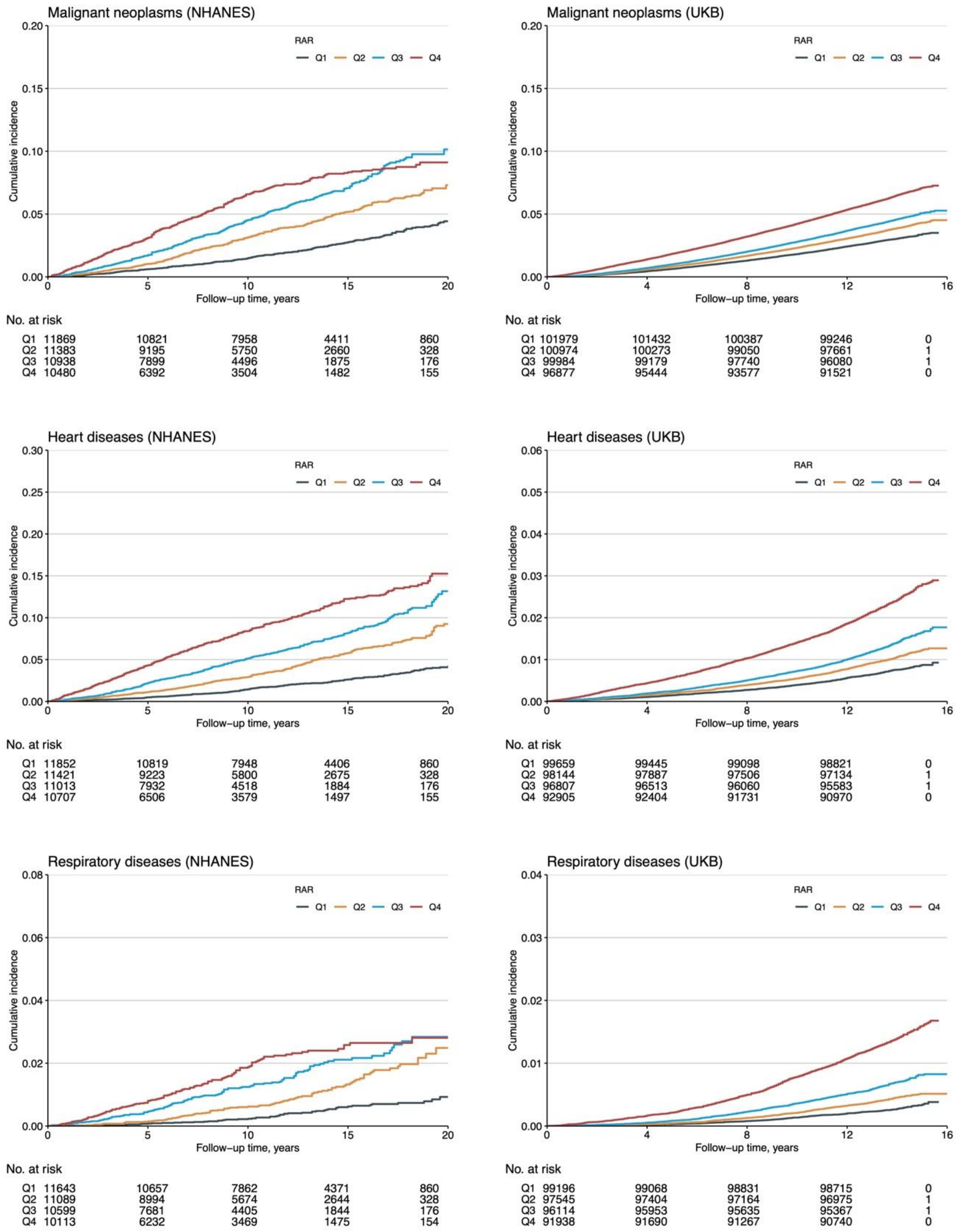

eFigure 3. Cumulative incidence of all-cause mortality according to quartiles of RAR in NHANES and the UK Biobank. RAR: ratio of red cell distribution width to albumin; HR: hazard ratio; CI: confidence interval.

© 2024 Hao M et al. *JAMA Network Open*.

eTable 1. Baseline characteristics of included and excluded population in NHANES

	Included Participants (N = 50622)	Excluded Participants (N = 6487)	P values
Age, years, M (SD)	48.57 (18.72)	50.77 (20.85)	<0.001
Gender, N (%)			0.009
Men	24486 (48.37)	3026 (46.65)	
Women	26136 (51.63)	3461 (53.35)	
Race, N (%)			<0.001
White	22277 (44.01)	2558 (39.43)	<0.001
Black	10366 (20.48)	1724 (26.58)	
Hispanic	13383 (26.44)	1490 (22.97)	
Educational status, N (%)			<0.001
College or above	24331 (48.06)	2757 (42.50)	
High school or equivalent	11270 (22.26)	1463 (22.55)	
Less than high school	13181 (26.04)	1959 (30.20)	
Alcohol drinking, N (%)			<0.001
Yes	32792 (66.64)	1767 (27.24)	
No	12701 (25.81)	895 (13.80)	
Smoking status, N (%)			0.003
Ever	22369 (45.45)	2730 (42.08)	
Never	26802 (54.46)	3544 (54.63)	
BMI, kg/m^2, M (SD)	28.86 (6.78)	28.52 (7.26)	<0.001
Death events	7590 (14.99)	1635 (25.20)	<0.001
Follow-up years	9.78 (5.56)	9.16 (5.89)	<0.001

© 2024 Hao M et al. *JAMA Network Open*.

eTable 2. Baseline characteristics of included and excluded population in UK Biobank

	Included Participants (N = 418950)	Excluded Participants (N = 83406)	P values
Age, years, M (SD)	56.56 (8.09)	56.40 (8.13)	<0.001
Gender, N (%)			<0.001
Men	193912 (46.29%)	35150 (42.14%)	
Women	225038 (53.71%)	48256 (57.86%)	
Race, N (%)			<0.001
White	395095 (94.31%)	77466 (92.88%)	
Non-White	23855 (5.69%)	5940 (7.12%)	
Educational status, N (%)			0.043
College or above	135232 (32.31%)	25867 (31.01%)	
High school or equivalent	207086 (49.48%)	38785 (46.50%)	
Less than high school	71628 (17.11%)	13626 (16.34%)	
Alcohol drinking, N (%)			<0.001
Yes	384483 (91.87%)	75751 (90.82%)	
No	33422 (7.99%)	7655 (9.18%)	
Smoking status, N (%)			<0.001
Ever	189097 (45.18%)	36862 (44.20%)	
Never	227735 (54.41%)	46544 (55.80%)	
BMI, kg/m^2, M (SD)	27.43 (4.78)	27.45 (4.93)	0.231
Death events	36793 (8.78%)	7707 (9.24%)	<0.001
Follow-up years	13.42 (1.99)	13.45 (2.13)	0.464

Two-sample Wilcoxon tests were applied for continuous variables, and chi-squared contingency table tests were applied for category variables.

© 2024 Hao M et al. *JAMA Network Open*.

eTable 3. Statistics of associations between RAR (continuous) and mortality

		Model 1			Model 2		
	**No. of events**	**HR (95% CI)**	**Z**	**P-value**	**HR (95% CI)**	**Z**	**P-value**
**NHANES**							
All-cause Mortality	7590	2.01 (1.96-2.07)	49.7	0	1.83 (1.76-1.90)	31.8	9.23E-222
Malignant neoplasm	1638	1.97 (1.84-2.10)	19.8	6.60E-87	1.89 (1.73-2.07)	14.0	9.48E-45
Heart disease	1961	2.17 (2.05-2.29)	27.3	6.75E-164	1.88 (1.74-2.03)	15.7	2.82E-55
Cerebrovascular disease	433	1.88 (1.64-2.15)	9.0	2.64E-19	1.35 (1.07-1.69)	2.6	0.00989459
Respiratory diseases	412	2.07 (1.82-2.35)	11.1	7.62E-29	1.99 (1.68-2.35)	8.1	5.98E-16
Alzheimer’s disease	283	1.70 (1.41-2.06)	5.5	3.27E-08	1.33 (0.98-1.81)	1.8	0.06590314
Diabetes	277	2.21 (1.91-2.55)	10.8	4.95E-27	1.55 (1.27-1.90)	4.3	1.98E-05
Other	2586	2.13 (2.05-2.23)	35.1	3.26E-269	1.97 (1.86-2.08)	23.6	2.87E-123
**UK Biobank**							
All-cause Mortality	36793	2.32 (2.27-2.36)	89.6	0	2.08 (2.03-2.13)	89.6	0
Malignant neoplasms	17657	2.14 (2.07-2.2)	48.8	0	1.93 (1.86-2)	48.8	1.05E-262
Heart disease	5358	2.57 (2.46-2.68)	44.7	0	2.42 (2.29-2.57)	44.7	3.54E-200
Cerebrovascular disease	1623	2.36 (2.16-2.57)	19.3	2.59E-83	2.15 (1.91-2.42)	19.3	2.28E-37
Respiratory disease	2636	2.84 (2.71-2.98)	42.3	0	2.96 (2.78-3.15)	42.3	9.93E-252
Alzheimer’s disease	687	1.32 (1.05-1.66)	2.4	0.01795501	0.85 (0.64-1.14)	2.4	0.27903753
Diabetes	253	3.18 (2.82-3.59)	18.7	3.22E-78	2.83 (2.35-3.4)	18.7	1.65E-28
Other	8579	2.5 (2.42-2.59)	53.6	0	2.4 (2.3-2.5)	53.6	0

RAR: ratio of red cell distribution width to albumin; HR: hazard ratio; CI: confidence interval; Z: z-statistic;

Model 1: Crude model; Model 2: Adjusted for age, gender, body mass index, race, educational levels, smoking status, alcohol consumption, hypertension, diabetes, heart disease, stroke, and cancer.

© 2024 Hao M et al. *JAMA Network Open*.

eTable 4. Statistics of associations between RAR (quartiles) and mortality in NHANES

	No. of events	HR (95% CI)	Z-statistic	P-value
**All-cause**				
Q1	1146	1 [Reference]	1 [Reference]	1 [Reference]
Q2	1732	1.16 (1.07-1.26)	3.67	2.39E-04
Q3	2072	1.42 (1.3-1.54)	8.29	1.17E-16
Q4	2640	2.21 (2.03-2.41)	18.21	4.68E-74
P for trend				< 0.001
**Malignant neoplasm**				
Q1	280	1 [Reference]		1 [Reference]
Q2	389	1.06 (0.9-1.25)	0.70	0.49
Q3	465	1.31 (1.1-1.55)	3.11	1.88E-03
Q4	504	1.85 (1.55-2.21)	6.72	1.84E-11
P for trend				< 0.001
**Heart disease**				
Q1	263	1 [Reference]		1 [Reference]
Q2	427	1.13 (0.96-1.33)	1.48	0.14
Q3	540	1.38 (1.17-1.63)	3.83	1.27E-04
Q4	731	2.22 (1.88-2.64)	9.21	3.21E-20
P for trend				< 0.001
**Cerebrovascular disease**				
Q1	70	1 [Reference]		1 [Reference]
Q2	122	1.09 (0.79-1.5)	0.50	0.62
Q3	113	1.04 (0.74-1.48)	0.25	0.81
Q4	128	1.3 (0.89-1.91)	1.36	0.17
P for trend				0.28
**Respiratory disease**				
Q1	54	1 [Reference]		1 [Reference]
Q2	95	1.43 (1-2.04)	1.96	0.05
Q3	126	2.04 (1.42-2.91)	3.89	1.01E-04
Q4	137	3.25 (2.21-4.77)	6.02	1.70E-09
P for trend				< 0.001

© 2024 Hao M et al. *JAMA Network Open*.

**Alzheimer's disease**				
Q1	52	1 [Reference]		1 [Reference]
Q2	73	0.81 (0.55-1.2)	-1.06	0.29
Q3	91	1.18 (0.8-1.74)	0.84	0.40
Q4	67	1.17 (0.74-1.85)	0.68	0.50
P for trend				0.27
**Diabetes**				
Q1	43	1 [Reference]		1 [Reference]
Q2	40	0.72 (0.45-1.15)	-1.39	0.16
Q3	82	1.34 (0.87-2.05)	1.33	0.18
Q4	112	2.25 (1.46-3.47)	3.67	2.4E-04
P for trend				< 0.001
**Other**				
Q1	384	1 [Reference]		1 [Reference]
Q2	586	1.27 (1.1-1.46)	3.30	9.65E-04
Q3	655	1.48 (1.28-1.71)	5.24	1.62E-07
Q4	961	2.67 (2.3-3.1)	12.97	1.71E-38
P for trend				< 0.001

RAR: ratio of red cell distribution width to albumin; HR: hazard ratio; CI: confidence interval; All models were adjusted for age, gender, body mass index, race, educational levels, smoking status, alcohol consumption, hypertension, diabetes, heart disease, stroke, and cancer.

© 2024 Hao M et al. *JAMA Network Open*.

eTable 5. Statistics of associations between RAR (quartiles) and mortality in UK biobank

	No. of events	HR (95% CI)	Z-statistic	P-value
**All-cause**				
Q1	5819	1 [Reference]	1 [Reference]	1 [Reference]
Q2	7622	1.13 (1.09-1.17)	6.83	8.33E-12
Q3	9276	1.26 (1.22-1.3)	13.27	3.43E-40
Q4	14076	1.82 (1.76-1.88)	36.23	1.83E-287
P for trend				< 0.001
**Malignant neoplasm**				
Q1	3055	1 [Reference]	1 [Reference]	1 [Reference]
Q2	3857	1.1 (1.05-1.16)	3.89	9.89E-05
Q3	4529	1.2 (1.15-1.26)	7.65	1.99E-14
Q4	6216	1.63 (1.56-1.71)	21.04	2.92E-98
P for trend				< 0.001
**Heart disease**				
Q1	735	1 [Reference]	1 [Reference]	1 [Reference]
Q2	1027	1.23 (1.11-1.35)	4.10	4.18E-05
Q3	1352	1.48 (1.35-1.62)	8.19	2.67E-16
Q4	2244	2.31 (2.12-2.53)	18.48	3.12E-76
P for trend				< 0.001
**Cerebrovascular disease**				
Q1	251	1 [Reference]	1 [Reference]	1 [Reference]
Q2	345	1.19 (1.01-1.41)	2.08	0.04
Q3	420	1.31 (1.11-1.54)	3.27	1.09E-03
Q4	607	1.8 (1.54-2.11)	7.41	1.29E-13
P for trend				< 0.001
**Respiratory disease**				
Q1	272	1 [Reference]	1 [Reference]	1 [Reference]
Q2	428	1.37 (1.17-1.6)	3.94	8.08E-05
Q3	659	1.87 (1.62-2.16)	8.45	3.01E-17
Q4	1277	3.5 (3.06-4.02)	18.02	1.39E-72
P for trend				< 0.001

© 2024 Hao M et al. *JAMA Network Open*.

**Alzheimer's disease**				
Q1	150	1 [Reference]	1 [Reference]	1 [Reference]
Q2	162	0.83 (0.66-1.04)	-1.62	0.11
Q3	181	0.83 (0.66-1.03)	-1.67	0.10
Q4	194	0.87 (0.69-1.08)	-1.28	0.20
P for trend				0.25
**Diabetes**				
Q1	22	1 [Reference]	1 [Reference]	1 [Reference]
Q2	39	1.71 (0.99-2.94)	1.93	0.05
Q3	55	2.26 (1.33-3.83)	3.02	2.5E-03
Q4	137	4.69 (2.88-7.64)	6.22	4.93E-10
P for trend				< 0.001
**Other**				
Q1	1334	1 [Reference]	1 [Reference]	1 [Reference]
Q2	1764	1.15 (1.07-1.23)	3.66	2.48E-04
Q3	2080	1.25 (1.16-1.34)	6.04	1.53E-09
Q4	3401	2.01 (1.88-2.15)	20.34	5.19E-92
P for trend				< 0.001

RAR: ratio of red cell distribution width to albumin; HR: hazard ratio; CI: confidence interval; All models were adjusted for age, gender, body mass index, race, educational levels, smoking status, alcohol consumption, hypertension, diabetes, heart disease, stroke, and cancer.

© 2024 Hao M et al. *JAMA Network Open*.
