# Supplementary material for: Ratio of Red Blood Cell Distribution Width to Albumin Level and Risk of Mortality
Source: JAMA Netw Open. 2024 May 28;7(5):e2413213. doi: 10.1001/jamanetworkopen.2024.13213 (PMC11134218; doi:10.1001/jamanetworkopen.2024.13213)
Supplement: Supplement 2. — Data Sharing Statement [file jamanetwopen-e2413213-s002.pdf]

Data Sharing Statement

Hao. Ratio of Red Blood Cell Distribution Width to Albumin Level and Risk of Mortality. *JAMA Netw Open*. Published May 28, 2024. doi:10.1001/jamanetworkopen.2024.13213

Data
Data available: No
